# Development of a core outcome set for paediatric upper limb spasticity; a structured review protocol for a systematic review of the literature and identification of a core outcome set using a Delphi survey

**DOI:** 10.1186/s13063-025-09141-w

**Published:** 2025-11-10

**Authors:** Liddle A, Sandhu K, Marcelline A, Swaby L, Ridsdale K, Dorman SL

**Affiliations:** 1 Department of Trauma & Orthopaedics, Sheffield Children’s Hospital, Sheffield, UK; 2 Sheffield Centre for Health and Related Research (ScHARR), School of Medicine and Population Health, University of Sheffield, Sheffield, UK

**Keywords:** Core outcome set, Delphi, Consensus methods, Upper limb spasticity, Children

## Abstract

**Background:**

Upper limb spasticity is a common condition in paediatric patients, often resulting from neurological disorders such as cerebral palsy, traumatic brain injury, or stroke. There are currently no standardised outcome measures to assess progress after interventions for upper limb spasticity in children. Wide variation in currently reported outcomes makes comparison of treatments difficult. This study aims to identify outcome measures that have previously been reported in studies evaluating the management of upper limb spasticity in children and to facilitate the development of a consensus core outcome set (COS) suitable for use in all future studies of upper limb spasticity in children.

**Methods/design:**

This study will include a systematic review of the academic literature to identify a list of outcome measures that have previously been reported. The list of outcome measures will be used in a consensus setting exercise with focus groups of key stakeholders to identify key outcomes. A Delphi process to include two rounds will then be used to define the most important outcomes to all stakeholders forming the COS.

**Discussion:**

Core outcomes represent the minimum expected data reported for a specific condition and will improve the quality of future studies, reducing bias, allowing easier comparison and enhancing opportunities for larger meta-analysis and more meaningful future research.

**Trial registration:**

Core Outcome Measures in Effectiveness Trials Initiative (COMET). Registered on 20 Jan 2024.

Prospero International prospective register of systematic reviews, registration number: CRD42024536296. Registered on 17 April 2024.

## Background

Upper limb conditions, particularly upper limb spasticity, impact a significant portion of the paediatric population. Globally, conditions like cerebral palsy affect 2 to 3.5 individuals per 1000 births [1]. Despite the prevalence of these conditions, the comparability of clinical trials assessing interventions is often hindered by the use of disparate outcome measures. A systematic review of paediatric orthopaedic literature, for instance, identified 2251 articles utilising 230 different outcome scales—half patient-reported and half surgeon-reported, with many lacking validation in children [2].

The meticulous selection of appropriate clinical assessments holds paramount importance in the design of controlled trials. This ensures comparability and guards against the distortion of benefits and harms [3, 4]. Moreover, for trial findings to inform policy and practice, the chosen outcomes must be relevant and significant to both patients and policymakers [5]. Consequently, there is a growing trend towards the development of core outcome sets (COS), standardised collections of outcomes tailored to a specific disease or population. A COS delineates the minimum set of outcomes that trials in a particular area should uniformly collect and report [3].

Notably, there is currently no established COS for clinical trials focussing on interventions for upper limb spasticity. As recommended by the guidelines established by the COMET [6] and COSMIN [7] initiatives, a crucial step in COS development involves identifying commonly used outcomes in the existing literature [3].

This systematic review aims to uncover such outcomes employed in studies investigating interventions for paediatric upper limb spasticity. It is anticipated that this will facilitate the development of a COS specific to upper limb spasticity in children through a Delphi process.

A COS for upper limb spasticity would help reduce heterogeneity in research allowing easy comparison of studies, improve accuracy of data interpretation, and reduce outcome reporting bias. Standardisation of outcomes will also lead to a reduction in omissions and increased statistical power for meta-analysis in this complex and difficult-to-assess patient group [8].

### Aims and objectives

The aim of this study is to develop a COS suitable for use in observational research, clinical trials, registries, and routine treatment of upper limb spasticity in children.

The specific study objectives are:

- To identify outcomes that have previously been reported in RCT, cohort studies, case–control studies and case series from a systematic review of the academic literature.
- To identify outcomes important to children and parents
- To prioritise outcomes from the perspective of key stakeholder groups using a two-round Delphi.
- To conduct a consensus meeting, compare outcomes considered important to all stakeholders and to integrate important outcomes into a combined COS.

## Methods/design

### Systematic review

The purpose of this study is to identify all outcomes reported irrespective of study quality. In addition, as there is no synthesis of outcome data from the included studies, a critique of the methodological quality of the studies is not necessary.

This process will be documented as per the Preferred Reporting Items for Systematic Reviews and Meta-Analyses (PRISMA) guidance [9].

Methodology similar to that reported in the development of COS for paediatric upper limb fractures will be used [10, 11].

#### Search methods for identification of studies

The search strategy will be applied to MEDLINE, the Cochrane Central Register of Controlled Trials (CENTRAL) and EMBASE. Multiple databases will be used to maximise the sensitivity of the search (January 2010 to August 2023).

The advantages conferred by using CENTRAL in addition to the other databases is that trials from other sources of research (e.g. journals not indexed in MEDLINE and conference proceedings) are hand-searched, and controlled trials from these are included. This improves the chances of identifying all relevant studies.

### Eligibility of studies

Two reviewers will independently screen all titles and abstracts of papers identified in the initial search. Titles of articles will be reviewed and included or excluded by using Rayyan [12]. A third reviewer will resolve any disagreements. Full-text manuscripts of any titles/abstracts that may be eligible for inclusion, or for which there is insufficient data in the title and abstract to make a clear decision, will be obtained.

The full-text papers will be assessed independently by two review authors, and any disagreement on the eligibility of included studies is resolved through discussion. Where resolution is not possible, a third review author will be consulted.

### Data extraction

The following data will be extracted from each study: paper and author details; year and journal of publication; study type; inclusion criteria (Table 1) and exclusion criteria; duration of follow-up; sample size; diagnosis and intervention(s) under investigation; primary and secondary outcomes; method of measurement; and time points at which outcomes were measured.

**Table 1:** Inclusion criteria for study selection

Study design	All study designs except systematic reviews, conference abstracts, case studies (< 10 cases) and expert opinion
Patient population	Study exclusively involving children (< 18 years), with upper limb neuromuscular disease or spasticity
Interventions	Any non-operative or operative intervention for management of upper limb spasticity
Outcomes	All outcomes
Other considerations	All studies must involve at least 10 cases of upper limb spasticity All studies must involve humans All studies must be in the English language

### Outcomes

The primary aim of the systematic review is to generate a list of all outcomes and measurement instruments reported historically in eligible studies.

### Data analysis and presentation

A comprehensive framework of health can be beneficial in developing a COS, favouring the content validity of the end product. A new framework has been developed that aims at including all key aspects of a health condition to ensure comprehensiveness of COSs.

Outcome terms will be assigned to one of the five core domains from the Dodd-Williamson classification (Table 2). The five core areas that should be covered by outcome measures to ensure a full breadth of reporting are: (1) adverse events, (2) death, (3) physiological/clinical, (4) life impact and (5) resource use [13].

**Table 2:** Overview of modified Dodd-Williamson [10] classification of outcomes

Core area	Core domains	Example
Adverse events	Adverse events	Unintended consequences
Death	N/A	N/A
Physiological/clinical	Musculoskeletal	Range of motion, tone, Tardieu
Life impact	Physical/social/role/emotional/cognitive functioning Health-related quality of life (HRQL) Delivery of care*	PROMS, activities of daily living, satisfaction
Resource use	Economic/hospital/need for intervention, societal burden	Length of stay, further surgery, physiotherapy
Technical considerations	Technical/surgical considerations	Motion analysis Radiographic angles

*Delivery of care does not refer to the resource delivery, but instead includes patient satisfaction, patient preference, adherence, withdrawal, tolerability

*PROMS* patient-reported quality of life, *N/A* not applicable

A sixth domain of technical consideration will be added for technical or surgical outcomes relevant to surgeons not covered by the existing framework [10].

Within each domain, we will evaluate the number of different outcomes used and the frequency of selection for each individual outcome measure. Where possible, we will also record the method of measurement and the time points at which they were measured.

### Identification of potential outcomes

A list of all potential outcomes will be identified from the systematic review as described above. Outcomes will be listed both individually and by domain to aid interpretation. All outcome domains and included outcomes will be reviewed by the Study Steering Group (SSG) to assess the suitability of the domain name and grouping. The SSG will consist of authors SD, AL, KR and LS.

To identify the outcomes of importance to all stakeholder groups, a Delphi approach will be used.

### Delphi process

The Delphi process is a structured, iterative method used to achieve consensus among key stakeholders in the development of a core outcome set (COS). It involves multiple rounds of anonymous surveys where participants rate the importance of various outcomes, with controlled feedback provided between rounds to refine opinions and move toward agreement.

**Fig. 1:**
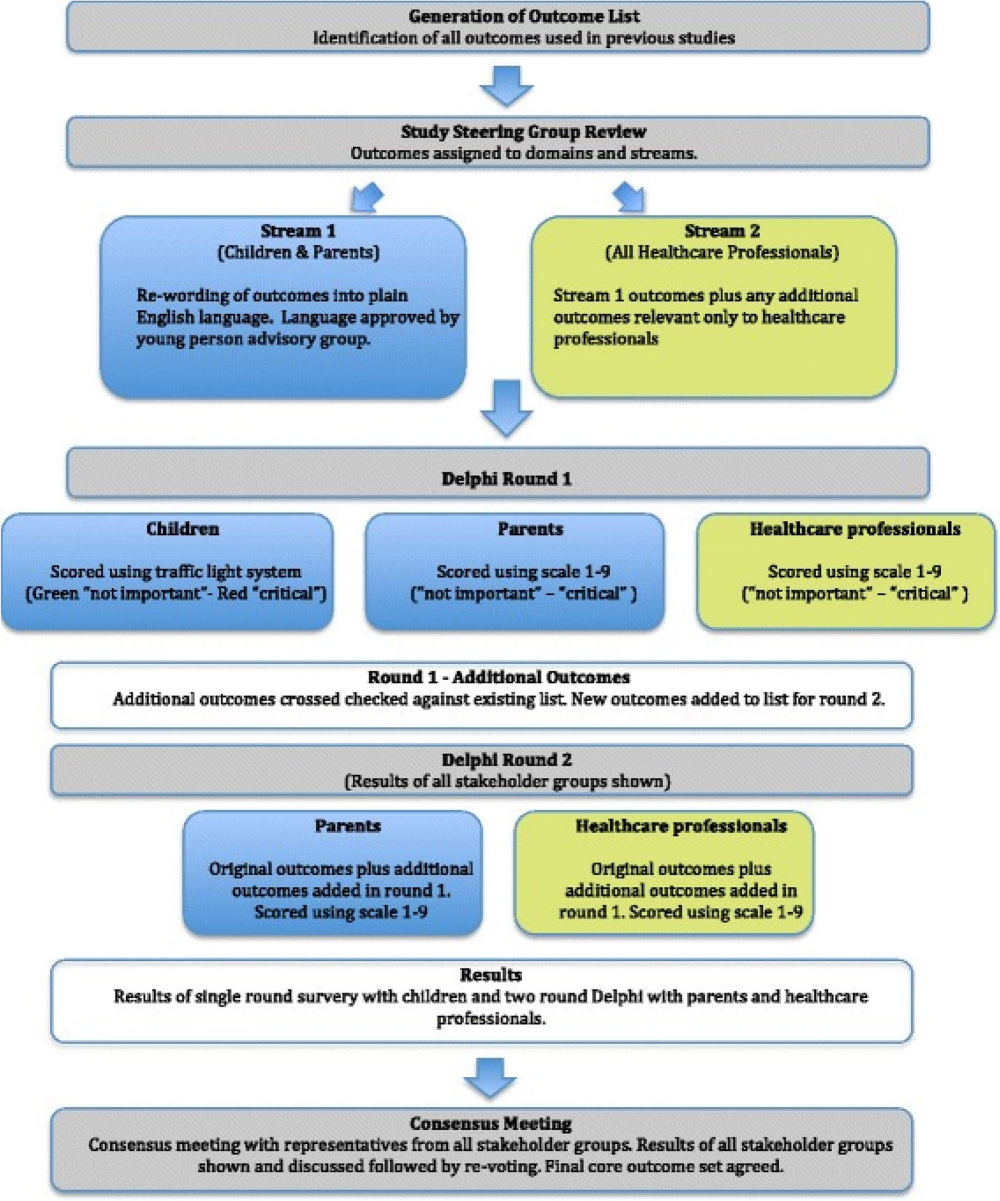
Overview of the core outcome set (COS) development process [10]

An overview of the COS developmental process is shown in Fig. 1. This will enable participants to provide anonymous opinions with equal influence given to all participants. This method also avoids individual participants’ responses being influenced by the opinion of their peers. Key stakeholder groups will consist of children, parents (Delphi stream 1) and clinicians (Delphi stream 2).

### Identification of outcomes of importance to patients and parents

It is essential that consideration is given to the opinions of parents and children regarding the treatment of upper limb spasticity. They should be given the opportunity to identify the most important outcomes and domains based on their own experiences and beliefs.

The opinions of children are key as they represent the group upon which the short- and long-term benefits and adverse effects of treatment will have the largest impact.

The most common cause of upper limb spasticity in children is cerebral palsy. Within this group, there is wide variation in physical and cognitive function. Children with good cognitive function who are able to understand and participate will be invited to interview. A minimum of 10 children will be recruited with no upper limit, and an equal spread of severity ranging from Manual Ability Classification (MACS) 1–5 will be included.

Demographic and contextual data will be collected for all participants, including age, sex, socioeconomic status, parental education level, spasticity distribution (e.g. unilateral vs. bilateral involvement, dominant limb), and functional classification using systems such as the Manual Ability Classification System (MACS). These variables will support subgroup analyses and help ensure the relevance and generalisability of the core outcome set (COS) across diverse populations.

Eligibility will be based on history of cerebral palsy or other neuromuscular disorder with upper limb spasticity. Children and families will require the ability to complete the interview in English without the need for a translator. Eligible children will be invited to attend a one-off interview structured by the use of a questionnaire. The authors feel this will improve compliance and accurate completion of the questionnaire. This will not be audio-recorded. It will consist of a researcher reading out the questions and recording the answers. The survey questionnaire (Delphi stream 1) has been designed to meet the developmental needs of a broad array of children using a traffic-light system for grading of outcome importance (green ‘not important’, amber ‘important but not critical’ and red ‘critical importance’. The traffic-light scoring system has been approved by a Young Person Advisory Group (YPAG) [10].

Children will also have the opportunity to add any additional outcomes they feel may have been missed. There will be no formal qualitative interview or qualitative analysis.

A separate sample of 20 parents will be identified from an existing cerebral palsy database at a UK tertiary paediatric centre. Parents will be invited to complete an online Delphi questionnaire (Delphi stream 1). Parents will complete all rounds of the Delphi process.

A plain English explanation for all outcome measures listed will be included in the stream-1 questionnaire for parents and children. Language will be approved by the YPAG.

This project is registered with the International Prospective Register of Systematic Reviews (PROSPERO), Core Outcome Measures in Effectiveness Trials (COMET), and has been subject to ethical approval (CRD42024536296). Informed consent and assent will be sought from participating children and families. Clinician consent will be assumed if participants agree to fill in the surveys.

### Identification of outcomes of importance to clinicians

#### Overview

The Delphi (stream 2) questionnaire will consist of the same outcomes used for children and parents, but additional technical and surgical considerations relevant only to clinicians will be added.

### Participants

The Delphi study will be conducted with clinicians who have a specialist interest in upper limb spasticity. Three clinician stakeholder groups will be surveyed, comprising UK surgeons, international experts with experience in clinical trials and Allied Health Professionals (physiotherapists, hand therapists and occupational therapists). Clinicians will only be invited to participate if they are currently involved in the clinical care of children with upper limb spasticity.

The inclusion of an international expert stakeholder group from a range of healthcare systems and perspectives will be included. Stakeholders will also have the opportunity to suggest additional outcomes they feel may have been overlooked by the literature review. This reduces the risk of any potential selection bias of outcomes within the English language literature.

Clinical leads for surgeons’ groups will be identified via the British Association for Upper Limb Spasticity, British Society for Surgery of the Hand and British Society for Children’s Orthopaedic Surgery. Participants are not required to have previous experience in clinical research. Eligible participants will be contacted via email and asked to complete an online Delphi questionnaire (stream 2). A minimum of 10 participants will be sought from the UK surgeons’ group and 10 from the group of international trialists. Where possible, 30 representatives will be sought from each stakeholder group.

The number of clinicians at each stage of the process will be recorded, including total number invited to participate; participants recruited to round 1; and numbers completing subsequent rounds. Attrition rates will be documented and analysed. Each participant will be given a unique registration number to enable tracking of attrition at each stage of the Delphi process. Reminder emails will be sent for those failing to complete each round.

Specialist software will be used to ensure that all information is recorded against the participant’s unique registration number only. Participants will not be able to access information about other participants or other individuals’ responses.

### Delphi survey

#### Delphi round 1

In the first round, the online questionnaire will also be used to request demographic information for registration. Information collected will include participant’s name, stakeholder group, clinical role, place of work and email address. Personal information will be stored in a separate database with a unique registration number.

At each stage of the Delphi process, participants will be given 3 weeks to complete the questionnaire. A reminder email will be sent at the end of week 2 to encourage completion and reduce attrition rates.

Participants who do not complete round 1 will be excluded from participation in further rounds.

### Round-1 survey format

All data will be collected using an online format. Content for round 1 will include the following: the participant demographics as outlined above, a list of outcomes to be scored, listed alphabetically and by domain. Participants will be asked to score listed outcomes and will have the option of adding any additional outcomes of importance not currently listed.

Participants will be asked the key question, ‘What outcomes may influence how you treat upper limb spasticity in children?’

The Grading of Recommendations, Assessment, Development and Evaluations scale will be used to score each outcome. Participants will be asked to grade each listed outcome in the format 1–9, with 1–3 deemed ‘not important’; 4–6 ‘important but not critical’ and 7–9 ‘critical importance’.

### Analysis of round 1

All additional outcomes proposed by participants will be reviewed by two assessors (SD and AL) to ensure that they represent new outcomes not already listed. In case of uncertainty or disagreement, a third assessor will be consulted.

The number of participants who scored each individual outcome will be recorded. The distribution of scores will also be summarised by the stakeholder group. All outcomes will be carried forward to round 2.

### Response rate in round 1

The response rate will be assessed and presented as follows: total number of participants registered; number by stakeholder group; number completing round 1; and the percentage of registered participants vs. invited based on information from clinical leads.

Continuation to round 2 will be determined based on response rate of round 1. In case of low numbers (< 10) the protocol for future Delphi rounds will be reviewed. Where responses do not differ greatly, an SSG review may suggest combining appropriate stakeholder groups.

### Delphi round 2

Round 2 data will be presented and recorded using an online format. Participants will be presented with data from all stakeholder groups. Data presented will include the number of respondents and distribution of scores for all listed outcomes and any additional outcomes added after round 1. Participants will also be able to view their individual score from round 1.

Children’s responses, scored using a validated traffic light system (green, amber, red), will be mapped to the GRADE scale as equivalent to scores of 3, 6 and 9, respectively. To ensure clarity, these responses will be presented in separate charts from other stakeholder groups to support interpretation and maintain the visibility of children’s perspectives in the consensus process.

Participants will then be asked to rescore the outcome in light of the additional information provided. New outcomes added in round 1 will also be scored. Any changes to scoring from round 1 will be recorded.

### Analysis of round 2

The total number of participants invited to participate in round 2 will be documented. The number of participants who scored each individual outcome will be recorded. The distribution of scores will also be summarised by the stakeholder group.

For each stakeholder group, each outcome will be classified as ‘consensus in’, ‘consensus out’ or ‘no consensus’ according to the consensus criteria (defined below).

Additional rounds of Delphi may be introduced if it is felt by the SSG that consensus had not yet been achieved.

### Consensus meeting

The final phase of the study will involve a consensus setting exercise by the Consensus Focus Group (CFG). The CFG will consist of representatives from all stakeholder groups (UK surgeon, Allied Health professionals, patient and parent), SSG and an independent COMET representative.

Results from round 2 of the Delphi survey will be presented and discussed, followed by voting to reach a final consensus COS.

### Definition of consensus

The classification of consensus (Table 3) will be used to determine whether a consensus has been reached or not for each individual outcome.

**Table 3:** Classification of consensus [14]

Consensus classification	Description	Definition
Consensus in	Consensus that outcome should be included in the core outcome set	70% or more participants scoring as 7 to 9 *and* < 15% participants scoring as 1 to 3
Consensus out	Consensus that outcome should not be included in the core outcomes set	70% or more participants scoring as 1 to 3 *and* < 15% of participants scoring as 7 to 9
No consensus	Uncertainty about importance of outcome	Anything else

In order to reach a consensus that the outcome should be included in the COS requires agreement by the vast majority (> 70%) that the outcome in question is of ‘critical importance’ with only a minority (< 15%) deeming it to be of ‘no clinical importance’ [14].

For an outcome to be excluded from the COS, the vast majority (> 70%) must score the outcome as of ‘no clinical importance’ with the minority (< 15%) of participants scoring it as ‘critically important’.

The threshold for the definition of consensus for this study has been predefined to prevent any bias of the end results towards beliefs of the research team.

In the event of a ‘no consensus’ outcome, the final decision will be made by the CFG. The structured expert CFG will enable representatives from key stakeholder groups to discuss differences of opinion, justify their perspectives, and make an informed decision using a Nominal Group Technique (NGT). It will be undertaken through a face-to-face meeting. Final consensus will be reached by means of a vote of stakeholders.

## Discussion

There is currently no published COS for assessment of upper limb spasticity in children. Multiple studies have demonstrated current outcome measures to be methodologically flawed, lacking in both validity and reliability in the assessment of upper limb spasticity in children [15–17]. There is a clear need to develop a more robust method of assessment of upper limb spasticity.

The development of COSs in this clinical area will improve the quality of future studies, reducing bias, allowing easier comparison and enhancing opportunities for a larger meta-analysis. It is also anticipated that this COS would provide useful information on core data to collect for use in upper limb spasticity registries.

## Data Availability

Data sharing is not applicable to this article as no datasets were generated or analysed during the current study.
