# Correction: Development of a core outcome set for paediatric upper limb spasticity; a structured review protocol for a systematic review of the literature and identification of a core outcome set using a Delphi survey

**DOI:** 10.1186/s13063-025-09304-9

**Published:** 2025-11-20

**Authors:** A Liddle, K Sandhu, A Marcelline, L Swaby, K Ridsdale, SL Dorman

**Affiliations:** 1 Department of Trauma & Orthopaedics, Sheffield Children’s Hospital, Sheffield, UK; 2 Sheffield Centre for Health and Related Research (ScHARR), School of Medicine and Population Health, University of Sheffield, Sheffield, UK

**Correction** **:** **Trials 26, 485 (2022)**

**https://doi.org/10.1186/s13063-025-09141-w**

Following the publication of the original article [1], we were notified that the second author’s last name was misspelled as Sandu instead of Sandhu.

**The original article has been corrected.**
